# Bandgaps of noble and transition metal/ZIF-8 electro/catalysts: a computational study†

**DOI:** 10.1039/d0ra02943b

**Published:** 2020-06-16

**Authors:** Alireza Baghban, Sajjad Habibzadeh, Farzin Zokaee Ashtiani

**Affiliations:** Chemical Engineering Department, Amirkabir University of Technology (Tehran Polytechnic), Mahshahr Campus Mahshahr Iran sajjad.habibzadeh@aut.ac.ir; Surface Reaction and Advanced Energy Materials Laboratory, Chemical Engineering Department, Amirkabir University of Technology (Tehran Polytechnic) Tehran Iran

## Abstract

Zeolitic imidazolate frameworks (ZIFs) are designed with metals as center atoms, connected by imidazole-like linkers. The created structures have been employed considerably in the field of advanced energy materials, including catalysis/electrocatalysis and energy storage and harvesting applications. In the present study, the bandgaps of pristine and doped ZIF-8 (using noble and transition metal dopants such as Pd, Pt, Ni, Mn, Co, Cu, Fe, and Ti) are determined. This can result in a promising approach to enhance the corresponding electronic properties while applying noble metal-free dopants. To determine the bandgap values, a quantum mechanical modeling based on density functional theory (DFT) was applied. Then, due to the time-consuming and complicated nature of this approach, the obtained results from the DFT study were then employed to develop the support vector machine (SVM) model to estimate the bandgap of the resulting nanostructure. The outcomes of the proposed model showed its high accuracy, with *R*^2^ of 0.98 and root mean squared error (RMSE) of 0.04. The developed model could have great value in designing various ZIF-8-based nanostructures, particularly when applied in electro/catalytic reactions, *e.g.*, electrocatalytic hydrogen evolution reaction or catalytic hydrogenation reaction, through a simple approach.

## Abstract 2

Band gap estimation for metal/ZIF-8 framework electro/catalysts by hybrid DFT and machine learning technique.

## Graphical abstract

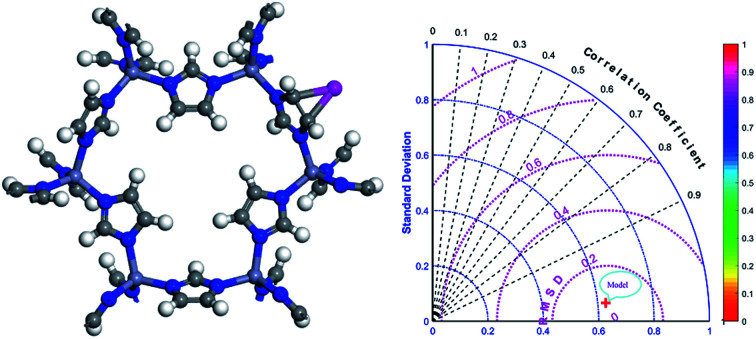

## 1. Introduction

Porous materials with tunable pore sizes, large pore volumes, and high surface areas have drawn much attention from researchers in recent decades.^1–5^ This field has gained even more significance with the development of an innovative type of porous material based on hybrid metal–organic frameworks (MOFs), which are well known as coordination polymers and hybrid organic–inorganic frameworks.^6–8^ MOFs with zeolitic structures, such as zeolitic imidazolate frameworks (ZIFs),^9,10^ include M–Im–M (M: transition metal cations, *e.g.*, Co, Zn, and Im: imidazolate linker), which can be synthesized by a self-assembly method.^11^ The different forms of ZIFs are topologically isomorphic with the conventional form of aluminosilicate zeolites. Namely, the M–Im–M angle is similar to the 145° Si–O–Si angle in zeolites.^12^ Among zeolitic MOFs, ZIF-8, referred to as Zn(2-methylimidazole)_2_ or Zn(mIm)_2_, is a highly crystalline porous material that possesses the pore diameter and accessible diameter of 1.16 nm and 0.34 nm, respectively.^13,14^

Applying ZIF-8 as an advanced energy material in energy harvesting, storage, and catalysis highlights the importance of the solid-state electronic properties of this porous structure.^15,16^ Particularly, the respective bandgap serves as one of the imperative parameters in the rational design of such materials.^17–19^ Information about this parameter can help researchers judge the performance of synthesized nanostructures in different applications, especially catalytic (*i.e.*, hydrogenation reaction) and electrocatalytic (*i.e.*, hydrogen evolution reaction). However, limited experimental data on the bandgap of such imidazolic crystalline structures have been reported in the literature,^20,21^ despite such experimental measurements becoming of great importance in designing new materials.^22^ Nevertheless, experimental measurements are quite costly and time consuming; therefore, theoretical studies based on quantum mechanical concepts with improved functionalities have been raised in the recent studies, which can potentially address the objective of bandgap determination.^23–28^

Computational material science, including DFT, Monte Carlo simulations, and molecular dynamics, can offer an opportunity to discover gaps in science and design new chemical structures and composites.^29^ Nonetheless, these computational approaches generate a huge amount of data, requiring the application of artificial intelligence (AI) in material data science. AI, a computational strategy that has been extensively applied in natural language processing, speech recognition, and computer vision, has recently been widely utilized for materials studies,^30^ specifically, in property prediction^31–34^ and prescreening in high-throughput materials search. Methods based on AI, such as decision tree, fuzzy logic, support vector machine (SVM), artificial neural network (ANN), *etc.*, are very robust tools used for DFT calculations, molecular dynamics (MD) simulations, and group contribution correlations. Such a combined approach between AI and material structural calculations can lead to a reduction of computational time.^35^

Machine learning patterns have emerged in solid-state electronic structure prediction based on fitting of DFT databases and utilization of different feature lists and network architectures.^36,37^ These strategies suggested a potential and alternative way for addressing the discussion of bandgap problems. Previous works on this issue employed regression or learning approaches. Namely, ordinary least squares regression (OLSR),^28,38,39^ sparse partial least squares regression,^39^ least absolute shrinkage and selection operator (LASSO),^38^ support vector regression (SVR),^40^ artificial neural networks (ANNs)^40,41^ and kernel ridge regression (KRR)^42,43^ have been used to determine actual bandgaps or bandgaps from computationally expensive beyond-DFT approaches. These employed DFT-KS bandgaps and constitutive elemental properties, including pseudopotential radii, first atomic ionization potential, electronegativity, atomic valence, standard melting points, atomic number, *etc.* Ferreira and Silveira developed multilayer perceptron (MLP) and extreme learning machine (ELM) artificial neural network (ANN) models to estimate bandgap values derived from DFT calculations for two-dimensional photonic crystals.^44^ Their results indicated the reliability of both ANN models, with the RMSEs of 0.7642 and 0.5627 for the MLP and ELM models, respectively. Moreover, another modeling study was carried out by Zhuo *et al.* to estimate the bandgap values of inorganic solids using the SVM method.^45^ They used 3896 experimental bandgap values extracted from the literature to construct the model. Moreover, Wang *et al.* developed a quantitative structure–property relationship (QSPR) model to estimate the bandgaps of metal oxide nanoparticles, which were obtained from DFT calculations.^46^ They used Vienna *Ab initio* Simulation Package to calculate the theoretical descriptors.

In this study, a combined quantum mechanical concept and machine learning approach was employed to estimate the bandgaps of noble and transition metal/ZIF-8 composites. In addition, a potential application of the optimized SVM model with GWO method was investigated. In doing so, we optimized the binding energy of metal/ZIF-8 structures, for which the corresponding bandgap values were determined by DFT simulation. The obtained bandgap values from DFT calculations were utilized to construct the SVM model in order to estimate the respective bandgaps with the simple variables.

## 2. Theory

### 2.1. Support vector machine (SVM)

One of the supervised learning methods, called SVM, was constructed by statistical learning theory for pattern recognition and data analysis, which is applied for data classification and regression.^47^ The aim of the present work is to use this algorithm as a regression approach to transfer the data to a high-dimensional space by employing a nonlinear function of *Φ*(*x*), and bring them to the first space. The mentioned nonlinear mapping is done based on development of the suitable kernel function of *K*(*x*_*i*_, *y*_*i*_).^48^ Furthermore, it is assumed that several points are not appropriately classified by a hyperplane; therefore, to overcome this challenge, the slack variable is employed.^49^ By considering that *m* data points exist in the data space and the training dataset of *D* = {(*x*_*i*,_*y*_*i*_)|*i* = 1, 2, 3,…, *m*}, a regression function can be introduced in accordance with *y* = *w*^*T*^*Φ*(*x*) + *b*, in which *Φ*(*x*) stands for nonlinear mapping function, and *b* and *w* stand for offsets and weight vectors, respectively. Therefore, the optimized equation for the support vector regression model is:1
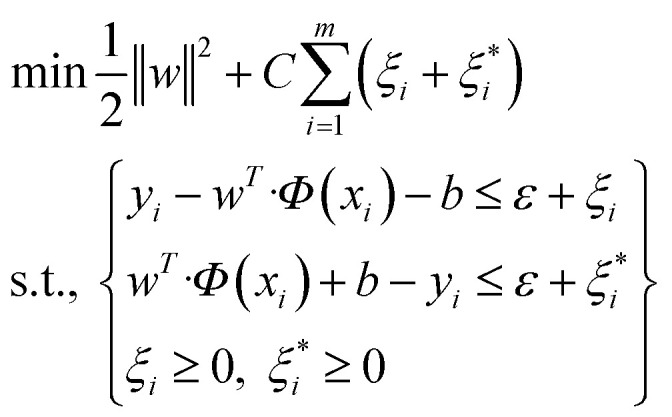
in which *C* represents the penalty parameter, *ε* shows the loss function parameter, and 
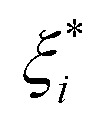
 and *ξ*_*i*_ represent the slack variables. The model loss is determined only when the absolute error of the predicted and real values is greater than *ε*.

The fundamental of this problem refers to a convex quadratic programming. To fuse the constraint into the cost function, the Lagrangian function is used, and the dual problem can be solved as follows:^50^2
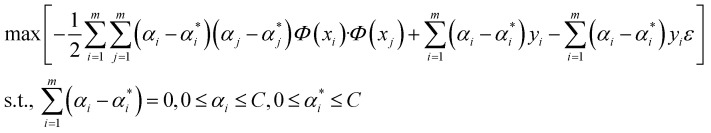


Herein, α is the Lagrangian multiplier, where the kernel function calculates the transformation relationship of the utilized dataset. In the present work, the radial basis kernel function is used:^51^3
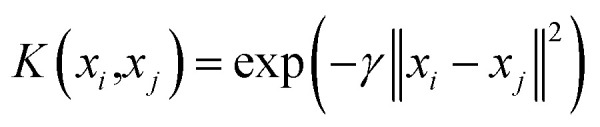
in which *γ* shows the RBF parameter.

According to the above discussions, there are two determining parameters in this training, namely, the penalty parameter *C* and the RBF parameter *γ*, referring to the generalization ability and estimation performance. Finally, it is worth mentioning that the hyper-parameters of SVM should also be optimized.

### 2.2. Gray wolf optimization (GWO)

The GWO algorithm is known as one of the optimization algorithms derived from simulation of the predation behavior and social hierarchy of gray wolf populations.^52^ A strict social hierarchy is considered for the gray wolf group, which can be demonstrated in a pyramid hierarchy. There are four grades in the gray wolf group based on the ranks. In these grades, the high-level wolf leads to the low-level wolf. During hunting, the activities, including aggression, encirclement, and capturing of the prey are conducted by the gray wolf group. Upon obtaining the best solution by GWO, the wolf group searches for the prey position, and then completes the search for the optimal solution based on the gray wolf fitness value and the relationship between the different grades.^53^

### 2.3. Density functional theory (DFT)

DFT rewrites the Schrodinger relationship to explain the conditions of electrons for a system.^54^ Hohenberg and Kohn developed this theory, in which all the ground-state properties are determined based on the charge density in which energy should be minimized. Such theorems proposed that there would be a potential to use it iteratively for improvement on an initial guess for the charge density. Later, Kohn *et al.*^55^ used the crystal structures to obtain a method for certain applications by reformulation of the basic equations to gather the most complicated interactions between electrons in an ‘exchange–correlation’ functional. It should be mentioned that the accurate form of the exchange-correlation functional has not been recognized, although there are some successful estimations by electron gas models and other extensions for several classes of materials.^56–58^ The inputs of a DFT calculation include the coordinates and identities of the atoms in the material within a repeating^59^ lattice, the exchange-correlation functional, parameters and algorithms for numerical and iterative convergence, and optionally, a method for more efficiently treating the core electrons in the system (for example, through the use of pseudopotentials). This is the point where DFT can generate structural information, including charge density and the electronic band structure, magnetic configuration, and binding energy.

## 3. Model development

### 3.1. Determining bandgap *via* DFT

**Fig. 1:**
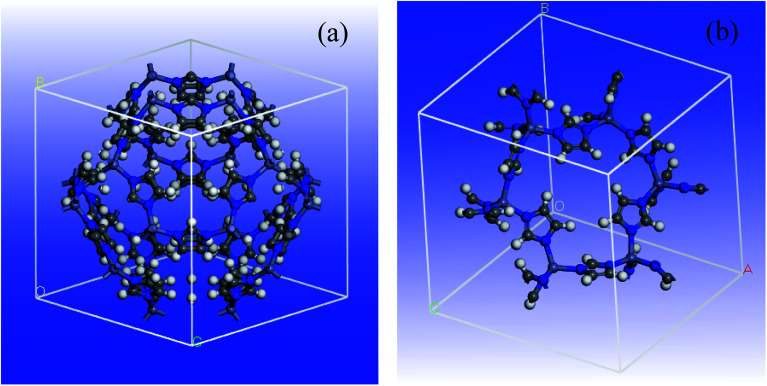
Schematic view of ZIF-8 MOF: (a) unit cell and (b) primitive cell.

A 3D cubic cell of ZIF-8 (see Fig. 1a) was constructed based on XRD pattern, and its primitive cell (shown in Fig. 1b) was used for subsequent calculations.

The determination of DFT combined by the generalized gradient approximation (GGA) were done in Dmol^3^ program.^60^ The Perdew exchange–correlation functional and double-numeric quality basis set were used.^61^ To treat the core electrons, DFT semicore pseudopotentials (DSPPs) were employed. The outputs showed that no magnetism was found by all the super-lattice models at their equilibrium lattice constants, though the spin-polarized determinations were done. In order to determine the energy and optimize the geometry, the convergence criteria were selected as follows: (a) a maximum displacement tolerance of 0.005 Å, (b) a maximum force tolerance of 0.002 Ha Å^−1^, and (c) an energy tolerance of 1.0 × 10^5^ Ha. In addition, an empirical dispersion-corrected density functional theory (DFT-D) developed by Grimme was employed to tackle the inappropriate description of the weak interactions by the standard PBE functional.^62,63^

**Fig. 2:**
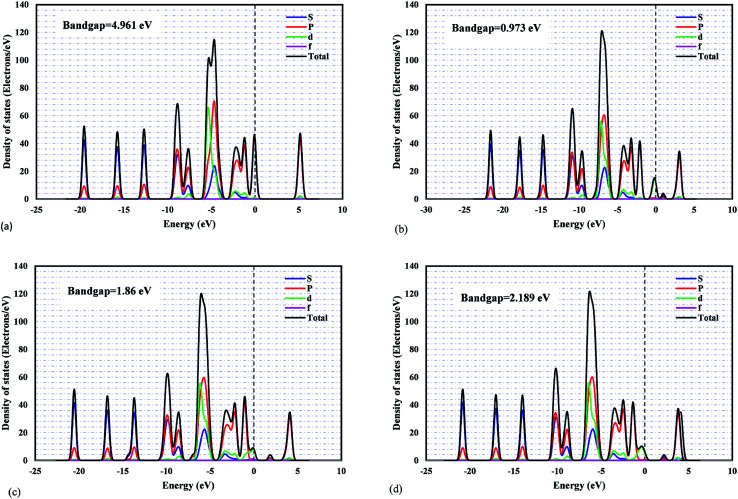
The PDOS plots for: (a) pristine ZIF-8, (b) Ni/ZIF-8, (c) Pt/ZIF-8, (d) Pd/ZIF-8.

The projected density of states (PDOSs) and the band structures of metal/ZIF-8 were calculated. This is to understand the orbital contributions and the respective electronic coupling. The bandgap value of pristine ZIF-8 is 4.961 eV, and based on electrochemical background, it is well known as a semiconductor material. Fig. 2 shows the projected density of states for pristine ZIF-8 and some important metal/ZIF-8 frameworks, such as Pt, Pd, and Ni/ZIF-8. Even though this figure includes the contribution from all orbitals, the orbital-projected DOS ascribes that the PDOS near Fermi energy originates mostly from the zinc d-orbitals for pristine ZIF-8. In addition, as can be seen, the bandgap values due to the addition of dopants were significantly decreased.

It was found that ZIF-8 has poor electronic conductivity.^64^ Thus, we tried to add precious metals and transition metals such as Pd, Pt, Ni, Mn, Co, Cu, Fe, and Ti as the dopants. Subsequently, the corresponding bandgap changes can be compared with the pristine ZIF-8. In addition, binary dopants, including Ni and other aforementioned metals, were also investigated within the metal composition range of 0–1.

**Fig. 3:**
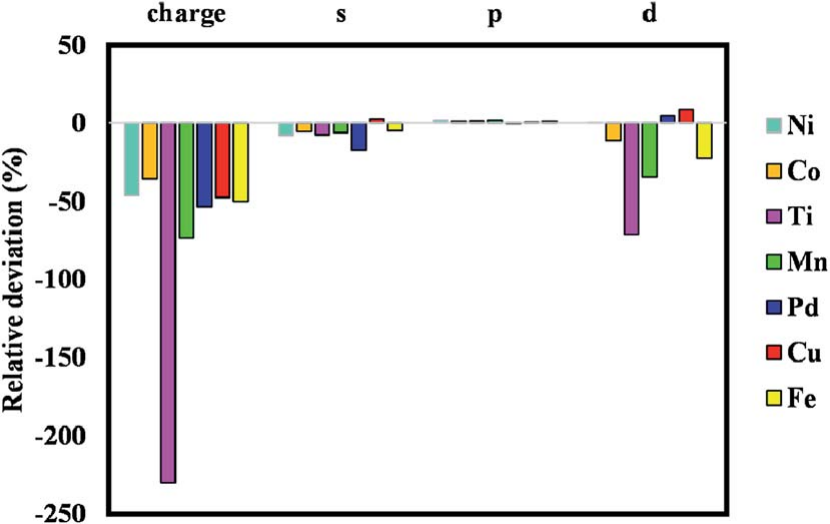
Relative deviation between obtained values from Mulliken population analysis for Pt/MOF and other metal/MOF composites.

Fig. S1 to S8† indicate the band structures and projected density of states for the addition of Ni, Mn, Co, Pd, Pt, Cu, Fe, and Ti atoms to the pristine ZIF-8 framework. As can be seen, the bandgap values were decreased significantly by the addition of Fe and Ti to this structure. The bandgap value can also lead us to find a suitable dopant for a certain application. Namely, considering catalytic hydrogenation or electrocatalytic hydrogen evolution reaction (HER), costly Pt-based electro/catalysts are considered as the most active materials. However, designing a cost-effective doped-ZIF-8 composite using the bandgap analysis can be of great value, where the bandgap values of such doped frameworks are close to that of Pt/MOF framework. Accordingly, the predicted metal dopant can be a good candidate for the hydrogen surface reaction application. As can be seen in Fig. S1 to S8,† the bandgap values for Pd, Cu, Ni, and Co metals are closer to that of Pt/ZIF-8. In addition, Mulliken population analysis confirms the above conclusion by comparing the charge for the *d*-angular momentum shell of the dopants. Fig. 3 indicates the relative deviation between obtained values from Mulliken population analysis for Pt/ZIF-8 and other metal/ZIF-8 composites.

**Fig. 4:**
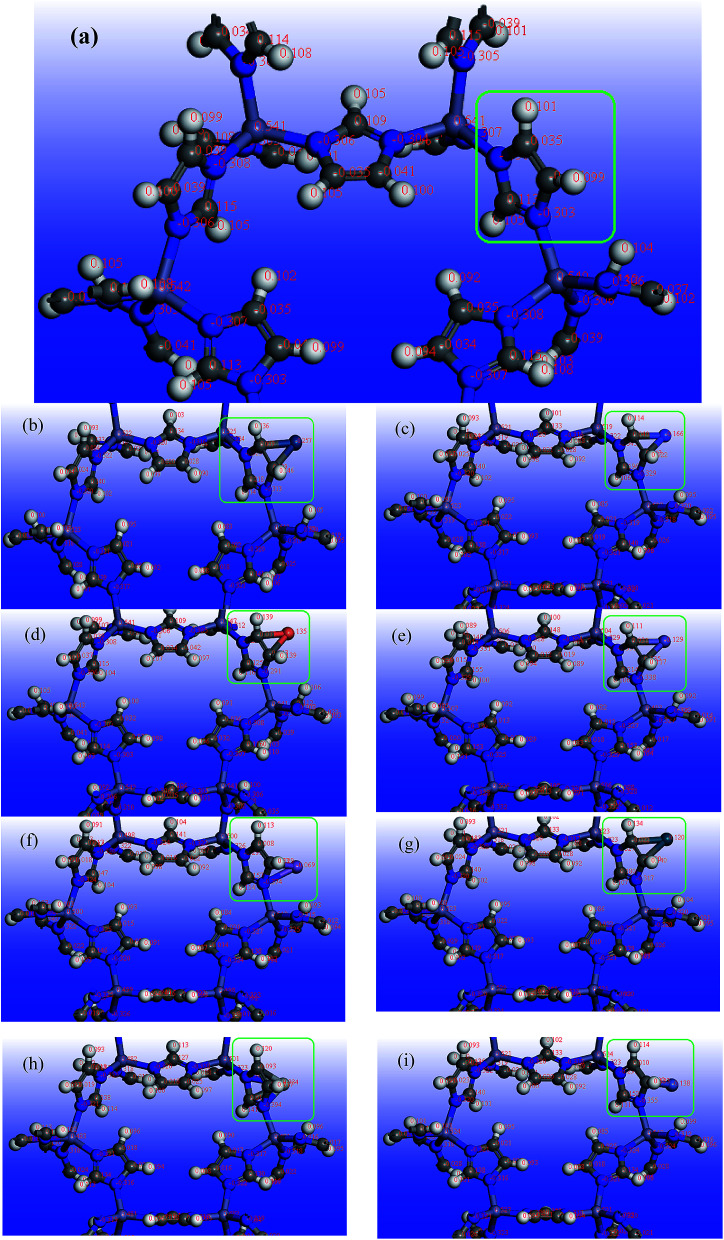
Schematic view of atomic charge difference with the addition of transition metals to ZIF-8: (a) pristine ZIF-8, (b) Pt/ZIF-8, (c) Co/ZIF-8, (d) Cu/ZIF-8, (e) Fe/ZIF-8, (f) Mn/ZIF-8, (g) Pd/ZIF-8, (h) Ti/ZIF-8, (i) Ni/ZIF-8.

Fig. 4 also illustrates the schematic of different metals doped on ZIF-8 and changes in atomic charge difference with the addition of these dopants to the pristine ZIF-8 framework.

### 3.2. Data used for the model construction

The results obtained from the DFT calculation were used to construct and test the SVM model. A total of 43 data points were employed; 70% of this data was used for training step, and the remaining 30% was applied to assess the generalization of the model. A portion of the dataset is presented in Table 1. The composition of binary dopants ranges between 0 and 1. The inputs of the proposed model are molecular weight, maximum energy level, number of electrons in s and d orbitals for both metal dopants, composition of the first dopant, and composition of the second dopant. All data were normalized within the ranges of −1 and 1 and fed to the SVM model.

**Table 1:** Bandgap values for some ZIF-8/metal composites determined by DFT calculation

Composite	Bandgap (eV)
ZIF	4.961
ZIF/Ni	0.973
ZIF/Co	1.241
ZIF/Co–Ni (50%)	1.237
ZIF/Cu	1.951
ZIF/Cu–Ni (50%)	1.393
ZIF/Mn	0.516
ZIF/Mn–Ni (50%)	0.984
ZIF/Pd	2.189
ZIF/Pd–Ni (50%)	1.394
ZIF/Pt	1.86
ZIF/Pt–Ni (50%)	1.392
ZIF/Ti–Ni (50%)	1.393

## 4. Results and discussion

### 4.1. Sensitivity analysis

A mathematical technique, *i.e.*, sensitivity analysis (SA), was applied to check the impacts of the input variables on the output. Additionally, there are other useful applications for SA, such as establishing the priorities of research, revealing technical errors, and identifying critical regions.^31,65^ In the literature, SA has two forms of analyses, local and global. Local sensitivity assumes that all the other inputs are set constant while the impact of one input on the target is evaluated.^66^ On the other hand, global sensitivity, as a typical method, studies the impact of the inputs on the target when all variables are varied.

**Fig. 5:**
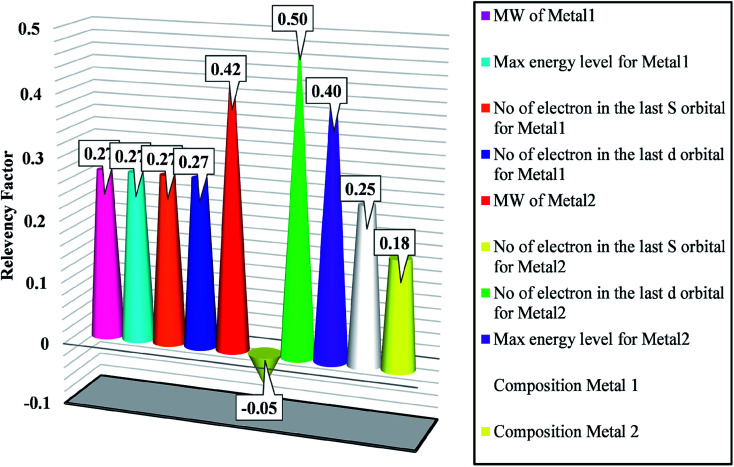
Sensitivity analysis for determining effective variables on bandgap.

The effectiveness of the inputs in GWO-SVM for bandgap prediction is shown in Fig. 5. As observed, the number of electrons in the last d orbital of the dopants shows the highest impact on the bandgap. In addition, characteristics of the first metal with the higher composition possesses a similar relevancy factor. The outcomes indicate that all of the designated inputs have crucial impacts on the bandgap values.

### 4.2. Designing GWO-SVM model

**Fig. 6:**
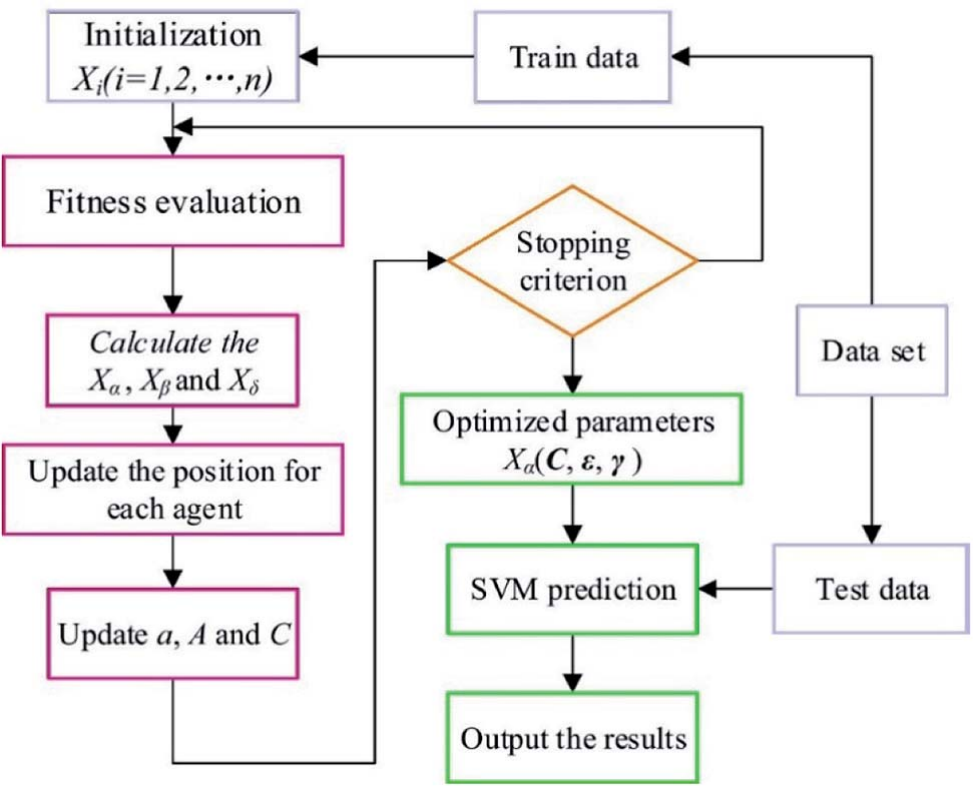
Flowchart of the GWO-SVM model.

According to the previous discussions, the performance of SVM algorithm is controlled by *C*, *ε* and *γ*. Therefore, in the present study, GWO was used to optimize these parameters. In Fig. 6, the scheme of the GWO-SVM algorithm is depicted.

The GWO fundamentally includes four parts: social hierarchy, tracking, encircling, and attacking prey steps. Four kinds of grey wolves, including alpha (*α*), beta (*β*), delta (*δ*) and omega (*ω*), are used for simulation of the wolf hierarchy, in which *α*, *β*, *δ*, and *ω* are solutions. The *α*, *β*, and *δ* are determined based on the corresponding fitness values, such that the best three solutions can determine the position of the prey. Detailed information explaining all parameters of this strategy can be found in the literature.^52^ As the final criterion is obtained, the GWO is stopped. Details of the proposed GWO-SVM model are summarized in Table 2.

**Table 2:** Details of the employed GWO-SVM algorithm

Parameter	Value/comment
Kernel function	Gaussian
No. of train data	37
No. of test data	6
Optimization technique	GWO
*C*	7525.674
*ε*	0.0358
*Γ*	0.00256

### 4.3. Outlier analysis

**Fig. 7:**
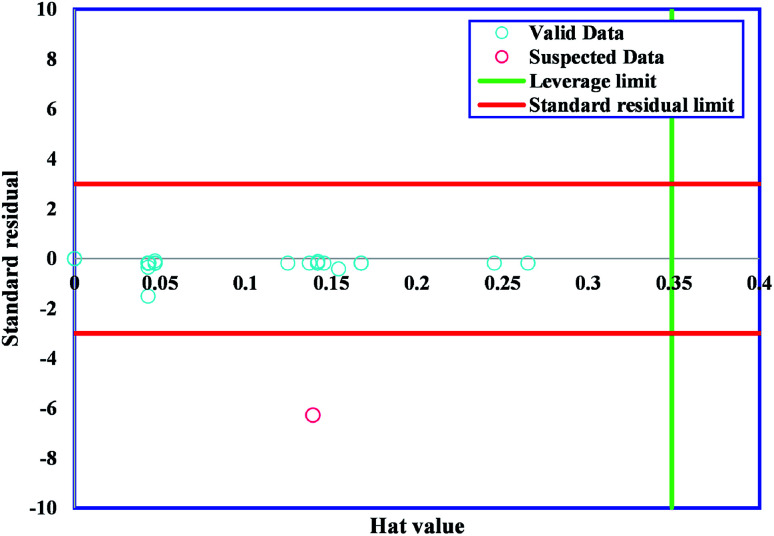
Williams plot of proposed GWO-SVM model.

One of the vital statistical approaches is outlier diagnosis, which is used to diagnose groups of data from the overall dataset. An efficient method, namely, leverage statistics, is applied for the detection of outliers.^67^ The critical Leverage limit (H*), the Hat indices (*H*), and the standardized (*R*) were considered in the present approach. The Hat index is expressed as follows:

4 *H* = *X*(*X*^*t*^*X*)^−1^*X*^t^,

where *X* and *t* are the two-dimensional (*n* × *k*) matrix and the symbol for transpose matrix, respectively. In this problem, the possible Hat solutions are on the main diagonal of *H*. The outliers are identified by depiction of the Williams plot. This plot expresses the correlation between the standardized residual and Hat index.^65^ A squared zone in the range of ±3 standard deviations and a leverage threshold of 3*n*/(*p* + 1) is defined as the valid zone of data (where *p* and *n* are, respectively, the number of input parameters of the model and the training points). The significant number of data placed in the ranges of −3 ≤ *R* ≤ 3 and 0 ≤ *H* ≤ H* shows that GWO-SVM can be applied in a wide domain. Outliers are defined as data (*R* and *H*) excess the ranges [−3, 3] and [0, H*], respectively. Fig. 7 shows the Williams plot of the outputs of GWO-SVM.

The majority of bandgap values studied in the present research lay in the reliable domain of [0, H*] and [−3, 3], except for one point placed in the range of *R* < −3, showing that the GWO-SVM algorithm is not only effective in statistics but could also raise the ability to reflect the intrinsic relationships between the bandgap value and input parameters.

### 4.4. Model evaluation

**Fig. 8:**
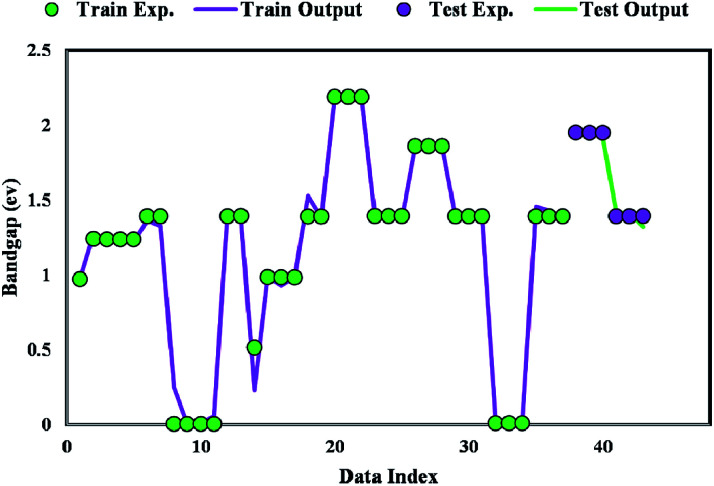
Simulated and predicted bandgap values obtained from the proposed GWO-SVM model.

Fig. 8 plots the bandgap value determined from the GWO-SVM model. In this plot, the obtained bandgap values are depicted *versus* the data index while illustrating the testing and training results. It can be seen that the proposed model can result in a great prediction capability.

**Fig. 9:**
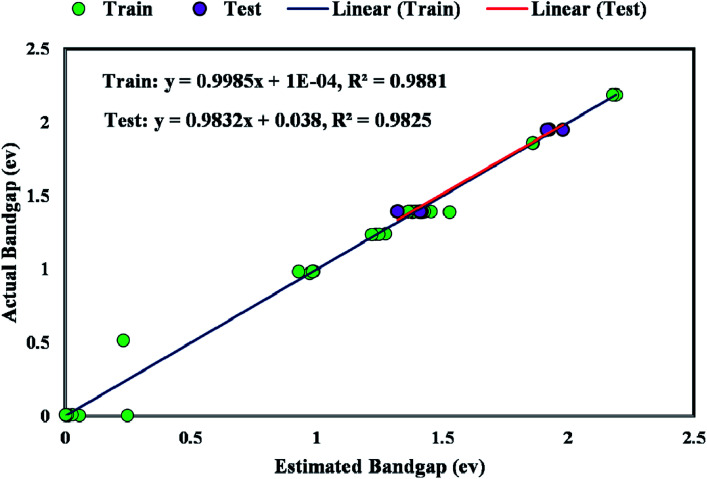
Regression plot between simulated and outcome of GWO-SVM model.

The coefficient of determination (*R*^2^) represents the proximity of determined values to real values. *R*^2^ varies in the range of 0 and 1.0. As this parameter gets closer to unity, the model predicts more accurately. Near-unity *R*^2^ for the developed model expresses its capability to determine the bandgap value of metal/ZIF-8 composites. As demonstrated in Fig. 9, the cross diagram of simulated and actual values illustrates an *R*^2^ coefficient of 0.9881 and 0.9825 for training and testing parts of the GWO-SVM models, respectively.

**Fig. 10:**
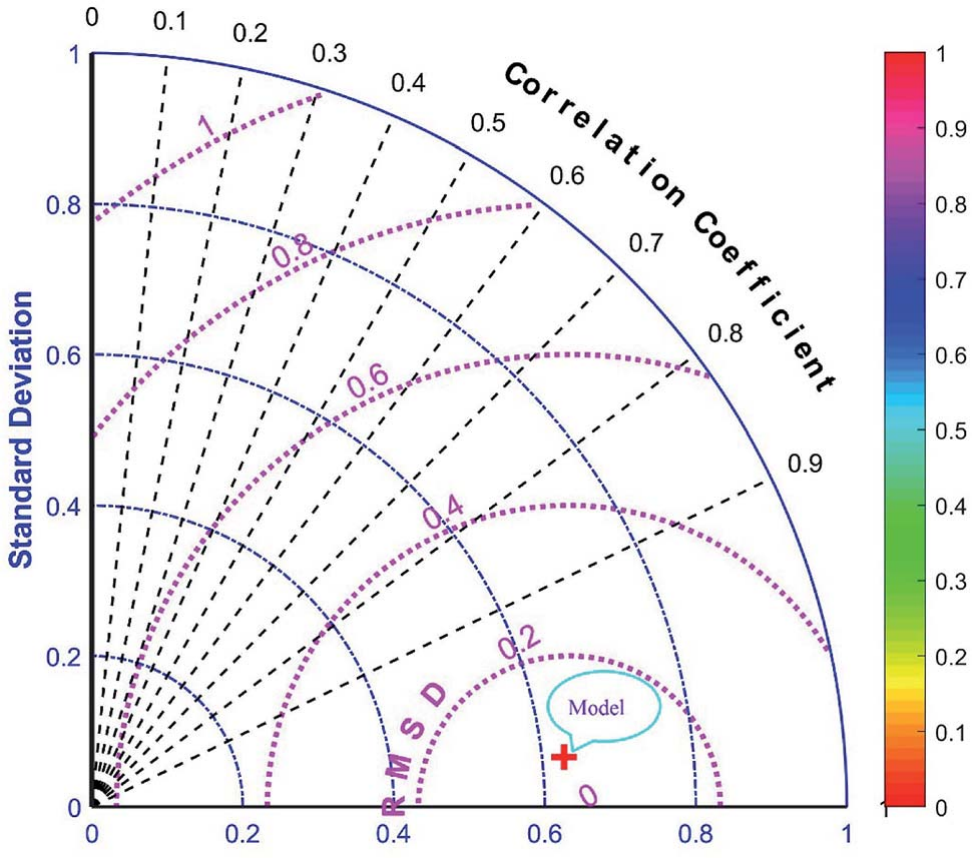
Taylor diagram of proposed GWO-SVM model.

**Fig. 11:**
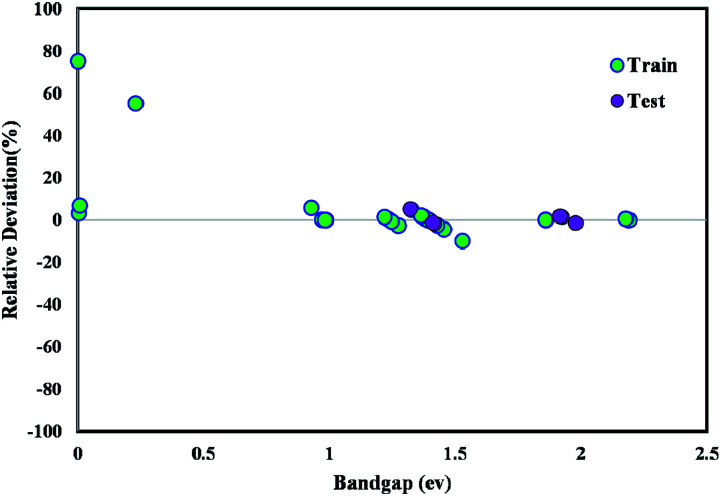
Relative deviation plot for the proposed GWO-SVM model.

The majority of bandgap values in both training and testing data sets are along the bisector line, which represents the precise determination of the GWO-SVM. Fig. 9 verifies the prediction capability and accuracy of the GWO-SVM model. The Taylor diagram of the proposed model is shown in Fig. 10 to statistically assess the model. The range in the color bar refers to the RMSE. An orange color is seen for the proposed model, indicating a value of RMSE of less than 0.1. According to the acquired values, GWO-SVM shows a superior accuracy with the minimum MRE, RMSE, and STD while having the maximum values of *R*^2^. Fig. 11 also illustrates the relative deviation percentage for the GWO-SVM model. It can be seen that the GWO-SVM model shows great precision, where the determined deviation does not exceed the 60% band.

### 4.5. Comparison with other methods

The obtained results from the proposed GWO-SVM model were statistically compared with two other machine learning methods, the adaptive neuro-fuzzy inference system (ANFIS) and the multilayer perceptron artificial neural network (MLP-ANN). The ANFIS approach was applied with five clusters and Gaussian membership function, in which its parameters were optimized by the particle swarm optimization algorithm. In addition, the proposed ANN model embedded five hidden neurons, where its parameters (weight and bias values) were tuned by Levenberg–Marquardt technique. Table 3 summarizes the statistical analyses obtained from all the applied models. It can be concluded from the statistical evaluations that the suggested GWO-SVM model can be regarded as the most accurate one.

**Table 3:** Comparison of different models

Model	*R*-squared	Mean relative error (%)	Mean square error	Root mean square error
GWO-SVM	0.982	2.14257	0.00148	0.03851
ANFIS	0.784	5.01616	0.01676	0.12947
ANN	0.942	3.47589	0.00832	0.09120

## 5. Conclusions

In this work, the bandgap of metal/ZIF-8 was determined, for the first time, by combining both concepts of quantum mechanics and machine learning approaches. A DFT study based on DMol^3^ code was carried out to determine the bandgap of the aforementioned composite, then the outcomes were used to construct the SVM model optimized by GWO algorithm. The inputs of the model were molecular weight, last layer, number of electrons in s and d orbitals for binary metal dopants, and composition of the first and second dopants. Based on the sensitivity analysis, the number of electrons in the last d orbital of dopants showed the highest impact on the bandgap. Moreover, according to the statistical analysis indicated by Taylor plot, the suggested GWO-SVM model showed a superior potential to accurately estimate the bandgap value. In addition, it was found from the Mulliken charge analysis that a relatively similar behavior was observed between the Pt-based composite and the other metal-based composites, with a close Mulliken charge. Hence, this study can effectively predict appropriate and cost-effective dopants for a certain application, particularly, hydrogen evolution reaction or hydrogenation reaction.

## Conflicts of interest

There are no conflicts to declare.

## Supplementary Material

RA-010-D0RA02943B-s001
