# Supplementary material for: Bandgaps of noble and transition metal/ZIF-8 electro/catalysts: a computational study
Source: RSC Adv. 2020 Jun 16;10(39):22929–38. doi: 10.1039/d0ra02943b (PMC9054687; doi:10.1039/d0ra02943b)
Supplement: RA-010-D0RA02943B-s001 [file RA-010-D0RA02943B-s001.pdf]

Electronic Supplementary Material (ESI) for RSC Advances.
This journal is © The Royal Society of Chemistry 2020

Band gap of noble and transition metal /ZIF-8 electro/catalysts: A computational study

Alireza Baghban[1], Sajjad Habibzadeh[1,2,*] Farzin Zokaee Ashtiani[2]

[1] *Chemical Engineering Department, Amirkabir University of Technology (Tehran Polytechnic), Mahshahr Campus, Mahshahr, Iran*

[2] *Surface reaction and advanced energy materials laboratory, Chemical Engineering Department, Amirkabir University of Technology (Tehran Polytechnic), Tehran, Iran*

Figure S1 to **Figure S8** indicate the band structures and projected density of states by addition of Ni, Mn, Co, Pd, Pt, Cu, Fe, and Ti atoms to pristine ZIF-8 framework. As can be seen, the bandgap values were decreased significantly by addition of Fe and Ti to this structure.

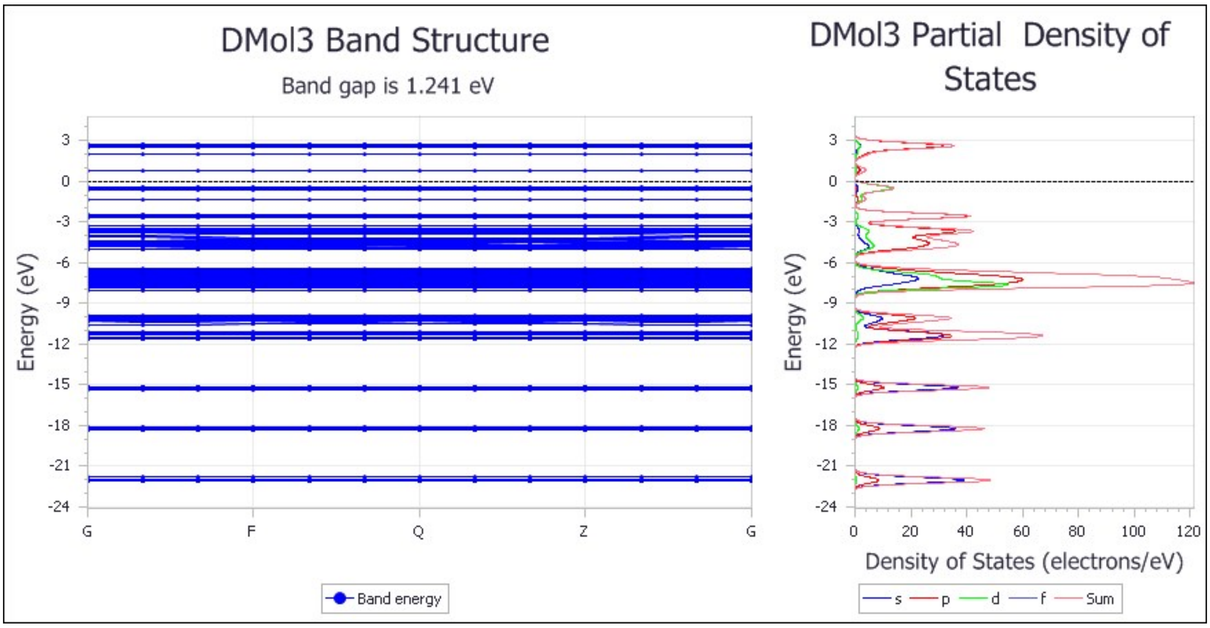

Figure S1: band structure and projected density of states for Co/ZIF-8

* Corresponding author:
Email: sajjad.habibzadeh@aut.ac.ir (S. Habibzadeh)

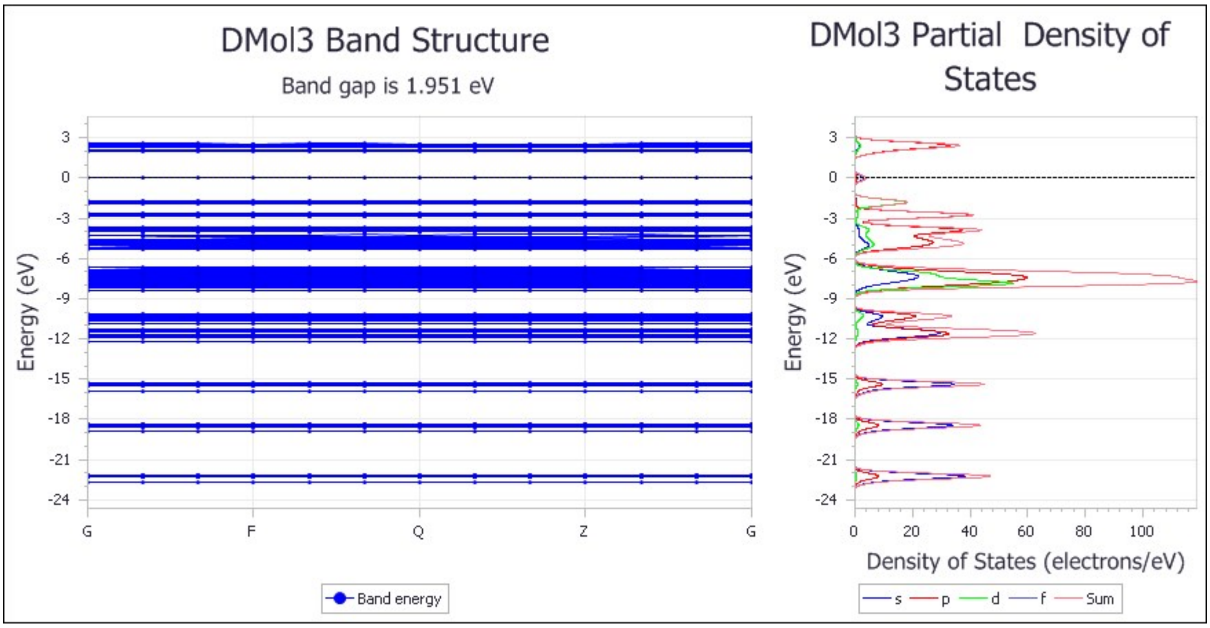

Figure S2: band structure and projected density of states for Cu/ZIF-8

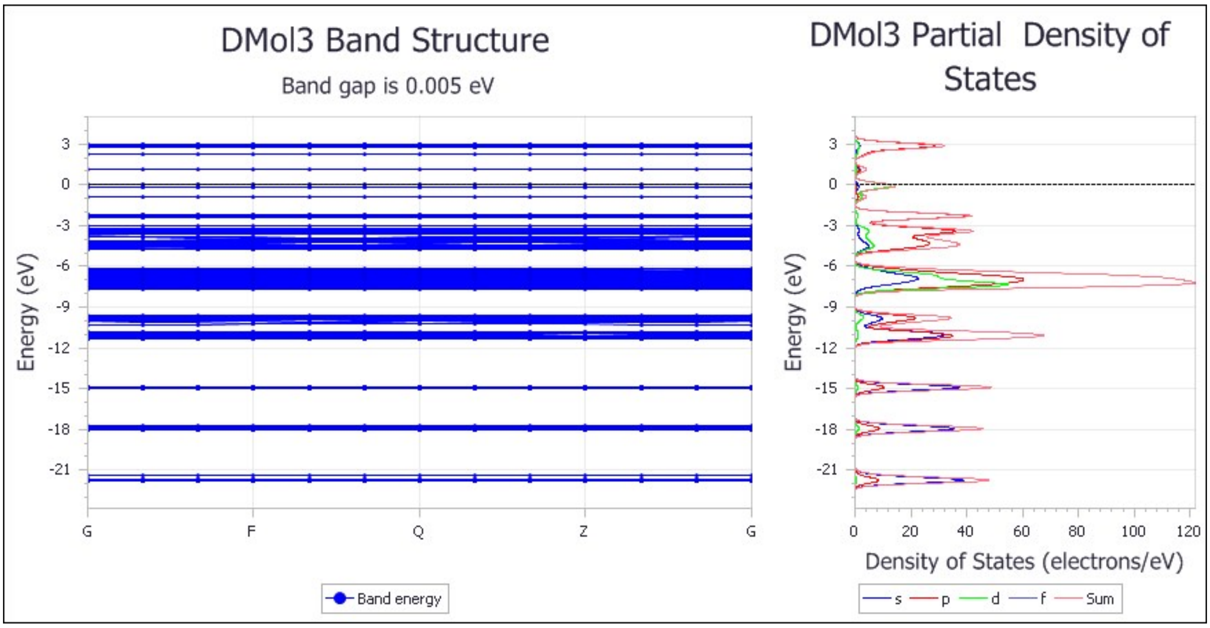

Figure S3: band structure and projected density of states for Fe/ZIF-8

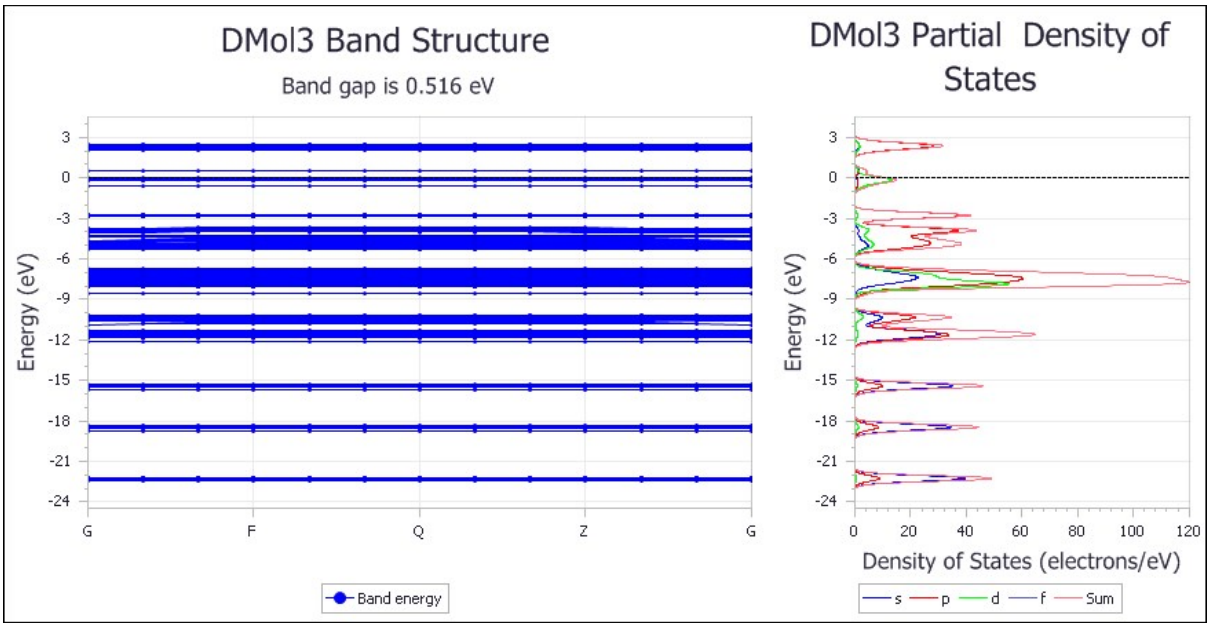

Figure S4: band structure and projected density of states for Mn/ZIF-8

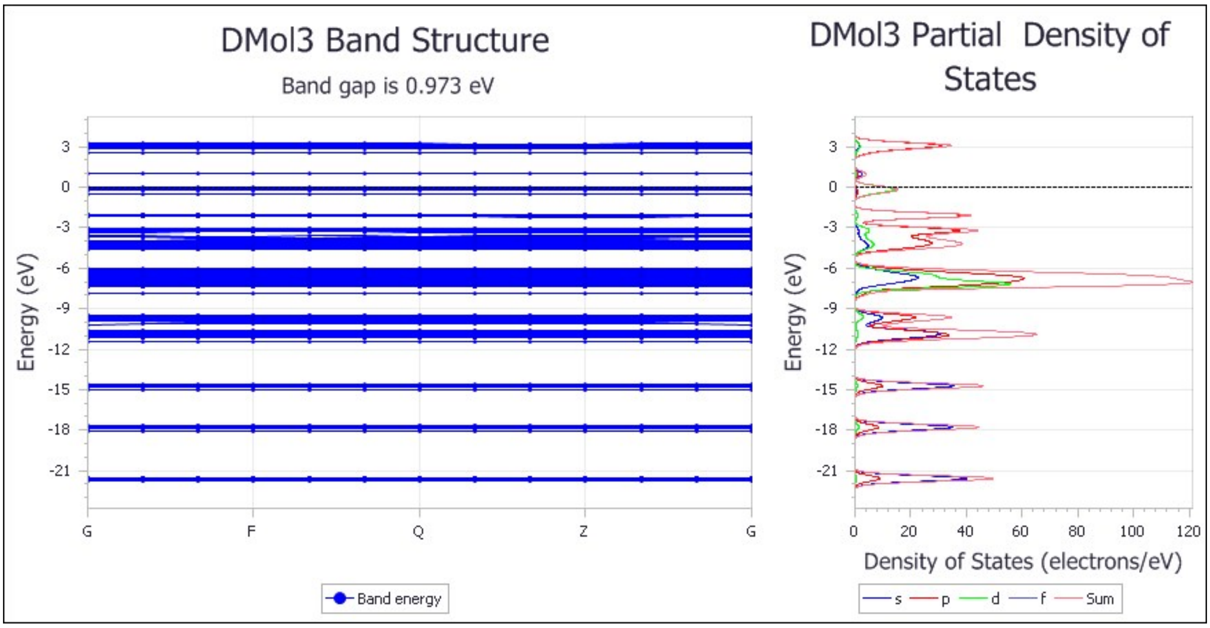

Figure S5: band structure and projected density of states for Ni/ZIF-8

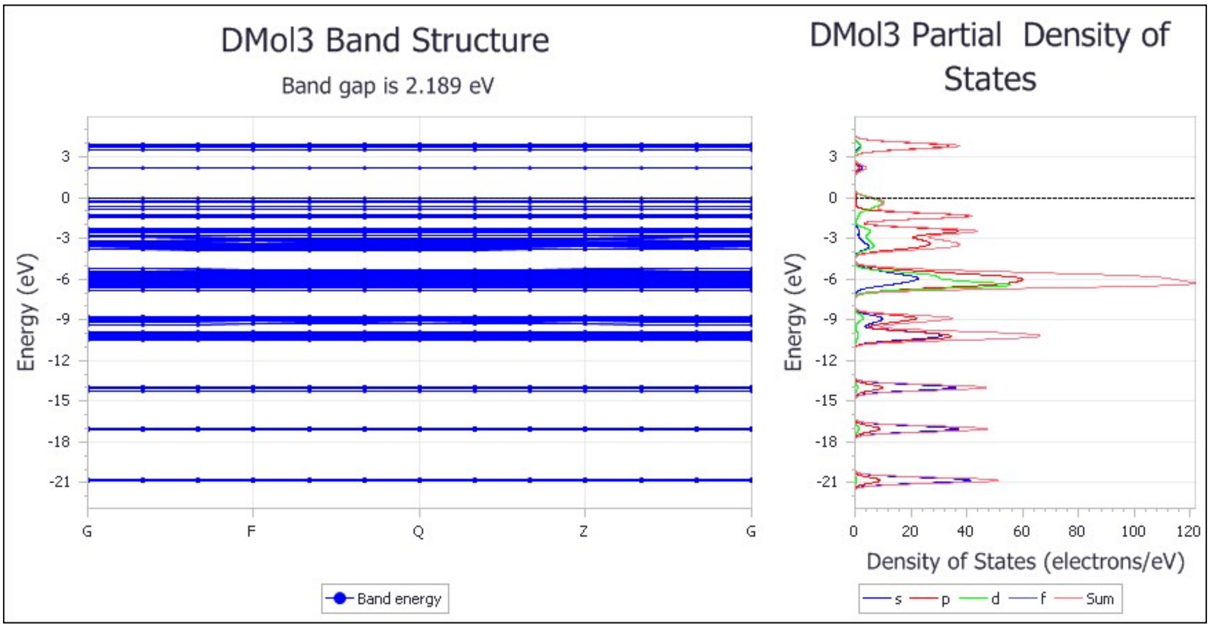

Figure S6: band structure and projected density of states for Pd/ZIF-8

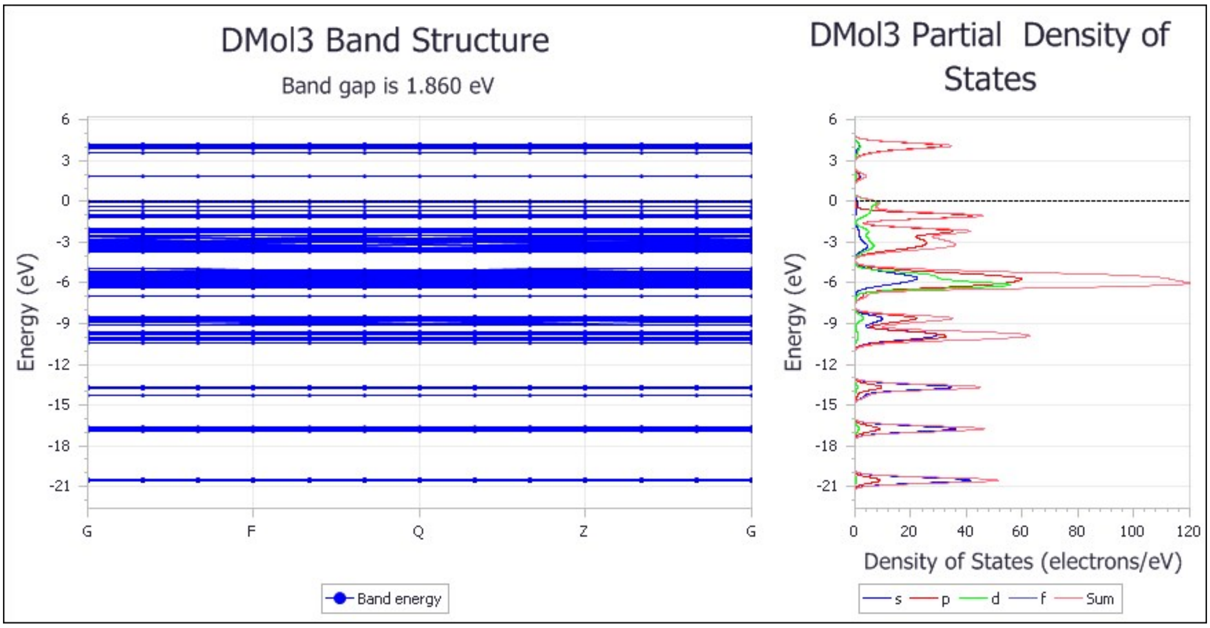

Figure S7: band structure and projected density of states for Pt/ZIF-8

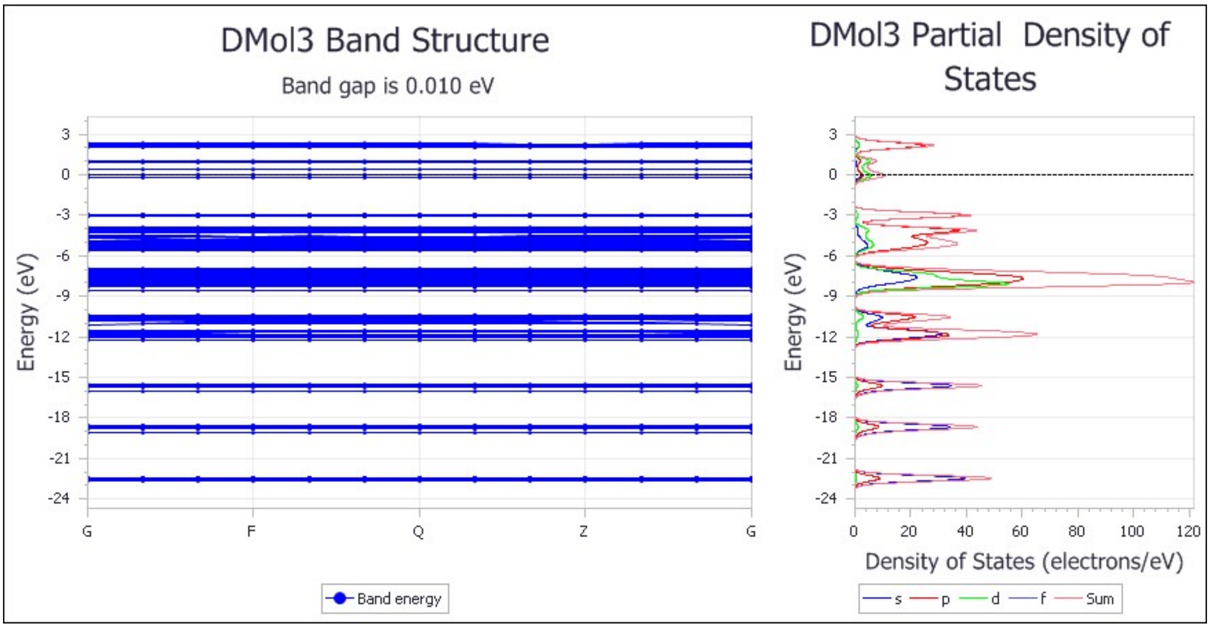

Figure S8: band structure and projected density of states for Pt/ZIF-8
